# EEG-Based Authentication Across Various Event-Related Potentials (ERPs)

**DOI:** 10.3390/s25164962

**Published:** 2025-08-11

**Authors:** Abeer Al-Nafjan, Lamia Alahaideb, Mashael Aldayel, Hessah Aljumah

**Affiliations:** 1 Computer Science Department, College of Computer and Information Sciences, Imam Mohammad Ibn Saud Islamic University, Riyadh 11432, Saudi Arabia; 2 Information Technology Department, College of Computer and Information Sciences, King Saud University, Riyadh 11543, Saudi Arabia

**Keywords:** electroencephalography (EEG), biometric authentication, event-related potentials (ERPs), convolutional neural network (CNN)

## Abstract

This study investigates the use of electroencephalography (EEG) signals for user authentication as an innovative approach to enhancing security within the cybersecurity domain. Motivated by the limitations of traditional authentication mechanisms, we explore the viability of brainwave patterns as distinctive biometric markers for verifying user identity. This research utilizes a publicly available EEG authentication dataset comprising recordings from 38 participants, with data elicited through paradigms designed to evoke P300 and N400 event-related potentials (ERPs). A rigorous methodological framework was employed, including signal preprocessing, ERP and power spectral density (PSD) feature extraction, and a comparative evaluation of multiple machine learning and deep learning classifiers, such as support vector machines (SVMs), random forests (RFs), and convolutional neural networks (CNNs). The proposed CNN model demonstrated superior performance, achieving 99% accuracy in the N400-Faces task, highlighting its effectiveness in discerning complex neural signatures associated with semantic and facial stimuli. The findings of this study substantiate the feasibility of EEG-based biometrics as a secure, noninvasive authentication modality and contribute to the advancement of resilient authentication frameworks.

## 1. Introduction

Authentication is the process of verifying and confirming the identity of an individual or system before granting access to a particular resource or specific information. An authentication method consists of three essential components: “something you know,” such as passwords, secret handshakes, and PIN codes; “something you have,” such as a physical key, a membership card, or a cell phone SIM card; and “something you are,” such as a fingerprint, brainwaves, or other biometric methods [1].

It is crucial to acknowledge that modern authentication systems contain vulnerabilities that can be exploited by potential adversaries. The first method, “something you know” authentication, is susceptible to interception using shoulder-surfing techniques. The second method, “something you have” authentication, can be compromised via the forgery or theft of identity cards or keys. The third method, “something you are” authentication, is not entirely immune to manipulation because adversaries may employ deceptive techniques to spoof the identities of legitimate users and circumvent the authentication process. For instance, someone could forge a fingerprint or copy another person’s voice to gain unauthorized access [2].

Although biometric-based authentication systems are not entirely impervious to spoofing attacks, they typically provide a heightened level of security compared to traditional methods. By leveraging unique biometric traits, these systems significantly enhance the robustness of authentication processes and increase protection against unauthorized access and hacking attempts. Nevertheless, security vulnerabilities persist, particularly due to the potential forgery or misuse of these distinctive traits. Consequently, there is an imperative for the development of novel authentication methods that offer superior reliability, robustness, and resilience against sophisticated attack vectors and emerging threat techniques [3,4,5].

The electroencephalogram (EEG) signal, which captures the electrical activity of the brain, has emerged as a promising biometric trait for various applications, including authentication [5]. This research aims to investigate the potential of using EEG signals as an innovative authentication method for the cybersecurity industry. Conventional authentication methods, such as passwords, are susceptible to security vulnerabilities, as previously discussed. EEG signals can be used for user authentication, as they use unique brain signals that are difficult to falsify, replicate, or steal. In addition to addressing previous biometric shortcomings, brainwave authentication benefits from the sensitive nature of these waves to a person’s emotional state, thereby reducing the risk of mimicking.

This study focuses primarily on the use of brainwaves as a biometric trait for authentication, a concept that has been supported by EEG-based authentication studies that demonstrate the adequacy of EEG-measured brain signals for user authentication purposes [4,6,7]. Among the most relevant contributions in EEG-based authentication, Arias-Cabarcos et al. [8] demonstrated the feasibility of using P300 signals within visual oddball paradigms to distinguish between users with high accuracy under controlled conditions. Similarly, Singh et al. [9] explored both spontaneous and evoked EEG signals, comparing various classification models across multiple cognitive tasks to assess biometric uniqueness.

Our approach integrates both event-related potentials (ERPs) and power spectral density (PSD) features within a convolutional neural network (CNN)-based framework, enabling the model to capture both temporal and spectral dynamics of EEG signals. This dual-feature integration is not commonly addressed in previous studies, which typically focus on a single feature type or use less advanced classifiers. Furthermore, we provide a comprehensive comparative analysis across multiple ERP tasks using a publicly available dataset, demonstrating superior performance—particularly in the N400-Faces task—compared to prior methods.

This paper is organized as follows: Section 2 provides background information on EEG signals, and authentication. Section 3 presents the framework of the proposed authentication-based EEG system. Section 4 details the implementation. Section 5 shows the results and discussion. Section 6 concludes the paper.

## 2. Background

This section aims to provide a comprehensive foundation for understanding EEGs. It offers a discussion of its methodology, frequency bands, and spatial localization of brain functions. Moreover, the section examines the critical role of authentication in contemporary security frameworks, reviewing traditional and advanced methods while emphasizing the transformative potential of using EEG signals for enhanced security.

### 2.1. Electroencephalograms

An EEG is a noninvasive electrophysiological recording method that records electrical activity in the brain using electrodes affixed to the scalp [10]. Its portability, cost-effectiveness, and efficacy have made it the predominant method for neural signal acquisition [11].

**Figure 1:**
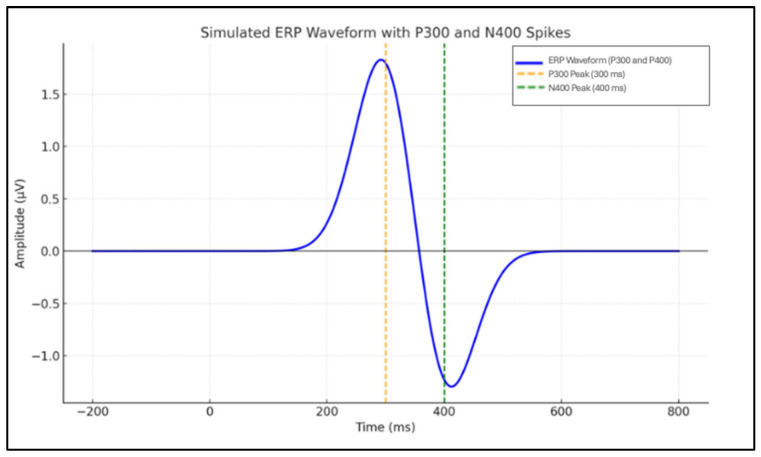
Simulated ERP waveform highlighting the P300 and N400 components.

ERPs are time-locked brain responses to cognitive or sensory stimuli. In this study, we focus on the P300 and N400 components due to their established roles in attention and semantic processing, respectively. These components are illustrated in Figure 1. The P300 is associated with attention and stimulus evaluation, while the N400 reflects semantic processing.

**Figure 2:**
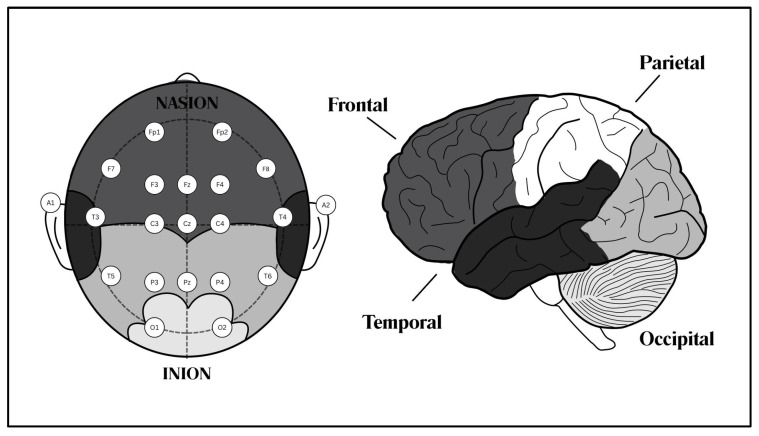
Electrode placement in the 10–20 standard and the corresponding brain lobes.

EEG data acquisition is conducted using low-impedance electrodes strategically positioned according to standardized configurations, such as the internationally recognized 10–20 system [12]. This systematic approach ensures consistency and reliability across studies, enabling precise recording of neural signals, which is essential for robust analyses and diverse applications. The electrode placement in the 10–20 system and the corresponding brain lobes associated with each electrode are illustrated in Figure 2, providing a visual framework for understanding the spatial organization and functional relevance of electrode locations. This standardization is instrumental in facilitating reproducibility and comparability in EEG research.

### 2.2. Authentication

Authentication is necessary for checking user identities and allowing only authorized access to systems, data, and resources [7]. This process involves verifying identity using methods such as passwords, biometrics, or cryptographic keys [13]. Using EEG signals for authentication systems is a big step forward in improving security and user experience, particularly where traditional methods are weak or unsuitable [14]. With the rise of complex cyber threats, improving authentication methods is vital to guarding against unauthorized access, data breaches, and identity theft and to providing a strong defense against various cyber dangers [1].

Authentication methods serve as pivotal mechanisms for verifying the identity of users accessing systems or resources. Two prominent approaches in this domain are single-factor authentication and two-factor authentication [15].

Single-factor authentication relies on a single category of credentials for user verification. Typically, this involves “something you know.” While single-factor authentication (SFA) offers a straightforward and easy-to-implement solution, it can be susceptible to security breaches, particularly in instances where passwords are weak, easily guessable, or compromised through unauthorized access.

Two-factor authentication (2FA) augments the authentication process by requiring users to present two distinct categories of credentials for verification. These categories typically include “something you know” and “something you have.” By necessitating the presentation of two different types of credentials, 2FA significantly enhances security compared to SFA alone. Even if one factor is compromised, the additional layer of authentication provides an added barrier against unauthorized access, thereby bolstering overall system security.

## 3. EEG-Based Authentication Framework

**Figure 3:**
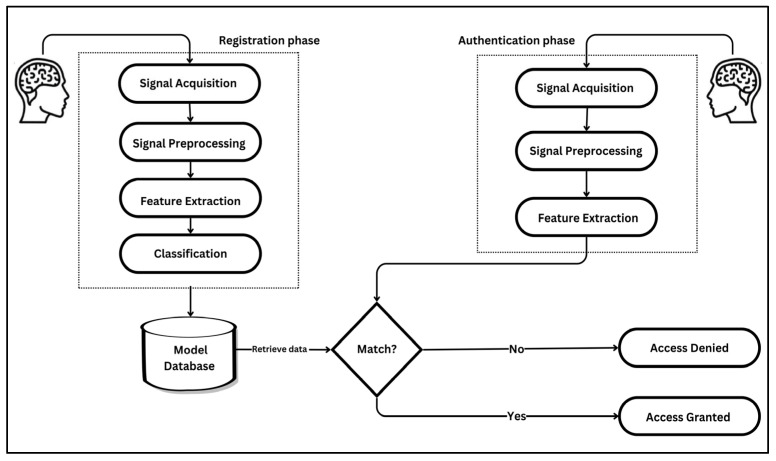
Flowchart describing an EEG-based system: registration and authentication phases.

The proposed architecture of the EEG-based authentication system in this study is structured into two phases: the registration phase and the authentication phase. The detailed flowchart outlining the sequential progression from registration to authentication within the EEG-based framework in Figure 3 provides a comprehensive overview of the workflow for both phases.

In the first step of the registration phase, brainwave signals from the user are captured via signal acquisition using EEG electrodes or channels, with data acquisition hardware recording electrical activity from the scalp. These raw EEG signals undergo data preprocessing, where preprocessing steps such as the bandpass filtering employed to minimize noise and artifacts, thereby enhancing the reliability of subsequent analyses.

Feature extraction follows, transforming preprocessed signals to extract essential information, guided by the literature to focus on ERPs and PSDs. Classification employs machine learning (ML) and deep learning methods to categorize and recognize patterns, with the database model storing structured user data for referencing during authentication.

Moving to the authentication phase, initialization marks the commencement of the authentication process through the frontend interface. Stimulus processing involves the frontend triggering an authentication request to the backend, which sends a stimulus back to the frontend for user presentation. Following stimulus presentation, the EEG headset captures the resulting brain signals and transmits them to the backend for further processing. Matching evaluates the similarity between real-time EEG data and stored model data, generating an authentication score. Finally, the result presentation relays the output to the frontend, providing the authentication outcome to the user.

The following subsections detail the sequential phases of the framework, from signal acquisition to classification, and provide an overview of the processes involved in transforming raw EEG data into actionable insights for authentication.

### 3.1. Signal Acquisition

The signal acquisition phase constitutes the foundational step of the proposed framework, facilitating the capture and recording of brain activity for subsequent analysis. Leveraging the noninvasive nature and high temporal resolution of EEG technology, this phase enables the detection of neural responses to cognitive and sensory stimuli with proven efficacy. Data acquisition is performed using specialized hardware, such as EEG headsets, that record electrical activity from the user’s scalp [7].

### 3.2. Signal Preprocessing

The raw EEG data, characterized by their continuous and unprocessed nature, pose significant challenges for direct interpretation due to their inherent complexity and susceptibility to artifacts and noise. To facilitate meaningful analysis and enable reliable comparisons across varying experimental conditions, preprocessing is an indispensable step in the EEG analysis pipeline. Preprocessing aims to enhance the signal-to-noise ratio, thereby ensuring a more accurate and robust assessment of neural activity [7].

Filtering is the most frequently employed signal processing technique in EEG research, serving as a foundational step to isolate relevant frequency bands and remove extraneous noise. Building on this, the current study utilizes bandpass filtering to eliminate low-frequency drifts and high-frequency noise [13].

### 3.3. Feature Extraction

The preprocessing stage effectively enhances the signal-to-noise ratio, ensuring the retention of high-quality neural signals and facilitating the accurate extraction of relevant features [9,16]. In this phase, meaningful information is derived from the EEG signals using ERP and PSD techniques, which emerged as the most frequently employed feature extraction methods.

ERP features capture the temporal dynamics of neural responses to specific stimuli, providing valuable insights into cognitive processes, such as attention and semantic processing. This approach is particularly effective for analyzing event-evoked neural activity, as evidenced by its extensive application in numerous studies [17,18,19,20]. ERPs are widely employed in authentication systems due to their ability to detect time-locked neural responses, reflecting underlying cognitive and perceptual mechanisms.

Table 1 provides an overview of commonly used ERP components and details their latencies, associated cognitive processes, and relevance to authentication. This highlights the ERP components’ critical role in leveraging neural responses to develop secure and reliable authentication mechanisms [21].

The ERP approach can enhance the performance of classification algorithms, particularly in high-stakes applications, such as user authentication, where robustness and accuracy are critical [17,18,19,20]. Meanwhile, PSD characterizes the frequency domain properties of EEG signals by quantifying the power distribution across various frequency bands. This technique is highly effective in identifying spectral patterns associated with cognitive and physiological states and offers robust features for classification tasks in authentication systems [13].

### 3.4. Classification

The extracted and selected features constitute the input for the classification models. Recent advancements have underscored the increasing significance of deep learning owing to its ability to model intricate nonlinear relationships and extract relevant patterns directly from raw data. By focusing on the most pertinent features, deep learning methodologies offer a robust framework for tackling complex problems, positioning them as highly effective tools in predictive modeling.

Given their proven effectiveness and widespread use in biometric authentication, the current study employed Support Vector Machines (SVMs), representing traditional ML, CNNs, and deep learning, for classification tasks. This selection is further justified by their frequent use in prior studies [8,17,18,19,20]. These models are particularly well suited for this application due to their robust capabilities to discern complex patterns within EEG signals and their established status as benchmark methodologies for evaluating authentication frameworks.

Additionally, we used a range of conventional machine learning algorithms, including RF, logistic regression (LR), gradient boosting (GB), decision tree (DT), and naive Bayes (NB), to provide a comprehensive comparative analysis. LR was employed as a baseline classifier. These models were selected for their complementary strengths in handling high-dimensional and nonlinear data.

## 4. Implementation

**Figure 4:**
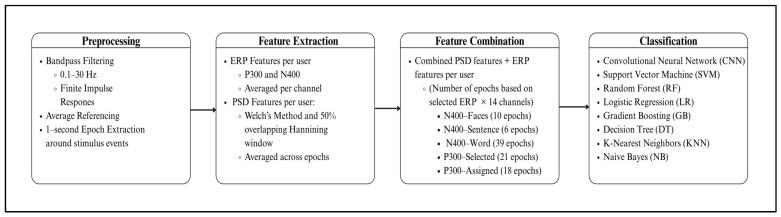
The research methodology.

This section details the implementation of the proposed EEG-based authentication system, encompassing the dataset utilized, preprocessing techniques, feature extraction methods, and classification models. The implementation leveraged a computational framework, employing Python 3 as the programming language, Scikit-Learn for machine learning processes, Scipy for signal filtering and preprocessing, MNE (Magnetoencephalography and Electroencephalography analysis software) for EEG-specific signal processing, and Keras for deep learning. The experiments were conducted on Google Colab Pro+, taking advantage of its high memory capacity to efficiently execute the deep learning models. Figure 4 illustrates the overall research methodology and system implementation workflow. It provides a structured overview of the major stages involved in the EEG-based authentication system.

### 4.1. Dataset Description

The Brainwave Authentication dataset [8] was used to enhance user authentication by applying advanced machine learning algorithms. This dataset was collected by recording participants’ brainwave activity using an Emotiv EPOC+ EEG headset [22] while they performed specific authentication tasks designed to generate distinct ERP brainwave patterns. This publicly available dataset comprises EEG recordings from 52 subjects, each subjected to five predefined stimuli-based tasks while being recorded for 20 min.

However, as clarified in the dataset’s official documentation, only 37 participants explicitly consented to public data release. Furthermore, one participant did not have any recorded events for the N400-Faces paradigm and was excluded from this experiment. Consequently, the final analysis was conducted on data from 36 participants.

The brainwave patterns collected from the participants are intended to serve as the foundation for developing authentication models based on EEG features, particularly the P300 and N400 responses. A summary of this dataset is presented in Table 2. Five protocols were used to elicit specific time-locked ERPs, namely the P300 and N400 components. The first two protocols targeted the P300 component, while the remaining three tasks were structured to investigate the N400 potential.

The oddball paradigm was used for P300, and the semantic processing paradigm was used for N400. In the oddball paradigm, participants were exposed to a randomized sequence of visual stimuli, including animal and food images, video clips, and grayscale images. The stimuli were categorized into target and nontarget stimuli using two distinct protocols: P300-Selected and P300-Assigned.

In the P300-Selected protocol, participants actively chose their target stimulus, whereas in the P300-Assigned protocol, the target was predetermined. To ensure sustained attention, the participants were instructed to perform an implicit counting task and silently count the occurrences of target stimuli.

In the semantic processing paradigm, participants silently read words that could have either expected or unexpected semantic relationships, aiming to trigger N400 responses associated with semantic processing using three distinct protocols: the word-pair task, the sentence context task, and the picture–word matching task. Table 3 provides an overview of the five acquisition tasks designed to evoke ERPs within the participants’ EEG recordings, namely P300-Selected, P300-Assigned, N400-Words, N400-Sentences, and N400-Faces.

### 4.2. Preprocessing

We implemented a preprocessing pipeline to enhance data quality by mitigating noise and artifacts. This stage was essential to ensure that the signals retained the neural activity of interest while reducing interference, aligning with best practices for preparing EEG data for advanced computational analysis. The preprocessing framework primarily employed bandpass filtering as a key signal processing technique. We applied a bandpass filter with cutoff frequencies of 0.1 Hz and 30 Hz to the raw EEG signals, similar to what was conducted in related research [18,23,24]. This filtering step retained neural activity within the physiological frequency range while eliminating low-frequency drifts and high-frequency noise. The zero-phase, non-causal band-pass filter was constructed using a Hamming-windowed Finite Impulse Response (FIR) design, providing a passband ripple of 0.0194 and 53 dB stopband attenuation. The lower transition band was set to 0.1 Hz (with a −6 dB cutoff at 0.05 Hz), while the upper transition band spanned 7.5 Hz (cutoff at 33.75 Hz). In addition to filtering, the EEG signals were re-referenced to the average across channels to reduce spatial noise.

To ensure the data were properly annotated for authentication purposes, we added a new column named “class” to the dataset, which assigned a unique label to each user. This label served as the target variable for the user prediction process, enabling the development of models to distinguish between users based on their EEG data.

### 4.3. Feature Extraction

The feature extraction stage transforms raw EEG epochs into meaningful representations suitable for downstream analysis. Epochs were segmented around stimulus onset to ensure temporal alignment with cognitive events, facilitating accurate ERP extraction. The continuous EEG signals were segmented into one-second epochs, spanning from 100 milliseconds prior to stimulus onset to 900 milliseconds post stimulus [17,18,19,20,25]. This temporal window was selected to comprehensively capture the ERP components, accounting for interindividual and intraindividual variability in peak latencies. Two primary categories of features were derived: ERP features and PSD features.

ERP features were computed per task and per channel by averaging across epochs, resulting in a two-dimensional matrix of shape (timepoints, channels). This approach preserved the full post-stimulus window (−100 to 900 ms), maintaining the integrity of early and late ERP components. ERP features were then derived by calculating the mean amplitude across the trials for each channel (*ch*) within the epoch. The calculation is shown in Equation (1), where $X_{ch}^{\left(i\right)}\left(t\right)$ is the EEG signal at time *t* for channel *ch* in the i-th epoch and N is the number of epochs.

(1) $$ERP_{ch}\left(t\right)\;=\sum_{i=1}^{N}\frac{1}{N}X_{ch}^{\left(i\right)}\left(t\right)$$

PSD features were extracted to characterize the frequency domain properties of the EEG signal, offering a complementary perspective on neural activity. Welch’s method over the 0.5–30 Hz range with a Hanning window was employed to compute the PSD for each channel within an epoch by segmenting the EEG signal into 50% overlapping windows and applying Fast Fourier Transform with a segment size of 256 samples.

The resulted bands were delta, theta, alpha, beta, and gamma across six selected channels, resulting in 30 spectral features (5 bands ×6 channels). Then, bands were averaged across epochs to obtain a matrix of shape (frequencies, channels) for each event. The calculation is shown in Equation (2), where $P_{ch}^{\left(i\right)}\left(F\right)$ is the PSD feature per channel (*ch*) at frequency *f* for the i-th epoch and N is the number of epochs.

(2) $$PSD_{ch}\left(f\right)\;=\sum_{j=1}^{N}\frac{1}{N}\;P_{ch}^{\left(i\right)}\left(f\right)$$

The resulting PSD estimates encapsulated the power distribution across frequency bins, providing a detailed representation of the spectral composition of the signal. This approach can enhance classification performance by capturing frequency-specific patterns in brain activity, which are vital for distinguishing individuals in authentication systems where precision and accuracy are paramount [25,26,27].

All features were extracted independently for each of the five experimental paradigms, thereby preserving task-specific neural dynamics. For example, to prepare the features for N400-faces, EEG signals were segmented over 10 epochs. For each epoch, PSD and ERP features were extracted from 36 users and 14 channels. The number of PSD features was 30 while the number of ERP features was 257, derived from $\left(10\;epochs\;\times \;36\;users\;\times \;14\;channels\right)$. Then, ERP and PSD descriptors were concatenated into a unified 287-dimensional vector per sample, comprising 257 temporal and 30 spectral features.

### 4.4. Classification

In this study, we proposed the use of CNNs for authentication and assessed their performance relative to conventional machine learning classifiers. A range of traditional machine learning algorithms (LR, RF, SVM, GB, DT, NB, and DT) were utilized.

**Figure 5:**
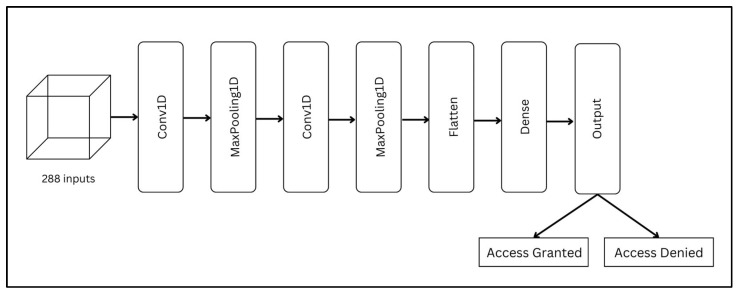
Architecture of the proposed CNN model.

Figure 5 shows the CNN architecture, which combines convolutional layers for feature extraction with fully connected layers for classification, making it suitable for complex sequence-based prediction tasks. The architecture comprises three consecutive 1D convolutional layers with 64, 128, and 256 filters, each followed by batch normalization and dropout rates of 0.2 for the first two blocks and 0.4 for the third. Max-pooling layers were included after the second and third convolutional layers. The fully connected layers consist of 256 and 128 units with ReLU activations, followed by dropout layers (rate = 0.3), and a final softmax output unit for multi-class classification.

The model was compiled using the Adam optimizer with a learning rate of 0.001 and a batch size of 16, and trained using early stopping (patience = 15) to prevent overfitting. Although the maximum number of epochs was set to 1000, training typically converged earlier. Weight initialization followed the normal distribution. The total number of trainable parameters was 250,546.

The 80/20 train–test split was applied to the number of trials with stratification to preserve class distribution. These configurations were selected based on a combination of preliminary experimentation and best practices reported in prior EEG classification studies [25,26].

The SVM used an RBF kernel with C = 1.0 and gamma set to ‘scale’. The random forest comprised 100 trees with Gini splitting and no depth limitation. GB was implemented with 100 estimators and a learning rate of 0.1. The KNN classifier used k = 5 and Euclidean distance. The DT relied on Gini impurity with unrestricted depth. The NB classifier assumed a GB of features. LR employed L2 regularization with a tolerance of 1 × 10^−4^ and the ‘lbfgs’ solver.

## 5. Results and Discussion

This section presents an evaluation and comparative analysis of the proposed EEG-based authentication system. The model’s performance was assessed, and the results were compared against various classical machine learning algorithms. The evaluation metrics include accuracy, precision, recall, the F1 score, and error rate metrics, specifically False Acceptance Rate (FAR), False Rejection Rate (FRR), and Equal Error Rate (EER). FAR refers to the proportion of unauthorized users incorrectly accepted by the system, while FRR denotes the proportion of genuine users who are mistakenly rejected. The EER represents the point at which FAR and FRR are equal, serving as a balanced indicator of overall system performance. The dataset was partitioned using a balanced holdout validation strategy, with 80% allocated for training and 20% reserved for final validation (testing).

The learning curves in deep learning are utilized to illustrate the progression of training accuracy in comparison to validation accuracy across epochs, as well as the evolution of training loss relative to validation loss over the same period. This analysis was conducted to ensure generalization capability to avoid issues of overfitting or underfitting. The degree of convergence between the training and validation accuracy curves serves as an indicator of the model’s generalization performance. A close alignment of the curves signifies robust generalization, whereas a persistent gap suggests potential overfitting, where the model excessively memorizes training data at the expense of its performance on unseen validation data. A consistent reduction in training loss reflects the effective optimization of the model’s parameters to minimize error on the training set. Conversely, the validation loss provides insights into the model’s ability to generalize to new, unseen data. A notable divergence between the training and validation loss curves, particularly an increase in validation loss after several epochs, serves as a clear indication of overfitting. Conversely, a steady alignment of the two curves over time demonstrates balanced learning and effective generalization.

Our model was designed to test and evaluate different types of ERP tasks, leveraging their diversity to assess their adaptability and robustness across various cognitive and neural patterns. This comprehensive approach not only enhances the diversity of ERP elicitation but also facilitates the development of authentication models that capitalize on both attentional and semantic neural markers, underscoring the potential of EEG-based biometrics for secure and reliable user identification.

### 5.1. CNN Model Performance Across Different ERP Tasks

This section presents the results obtained from the implementation of CNN models across various ERP tasks. The proposed CNN model demonstrated better performance in the N400-Faces ERP task compared to the other ERP tasks evaluated in this study, achieving an accuracy of 99%, and low ERR of 2%. These results highlight the model’s robustness in identifying and classifying relevant neural patterns associated with facial stimuli. Such outcomes underscore the model’s capacity to effectively process complex EEG data and extract meaningful features specific to this task.

In the P300-Assigned condition, the model yielded 93% accuracy, with an ERR of 6.4%. Similarly, the P300-Selected task demonstrated a performance profile of 92% accuracy, with associated ERR values of 7.2%, which is indicative of consistent reliability in the P300 component.

The N400-Words task yielded slightly lower performance, attaining 90% accuracy, accompanied by an ERR of 9%. While still within an acceptable range, these metrics suggest modest challenges in capturing the semantic processing of individual words. In contrast, the N400-Sentence task recorded the most limited performance across all dimensions. The model achieved 76% accuracy, with an ERR of 21.4%, indicating substantial difficulty in generalizing over complex linguistic structures at the sentence level, potentially due to increased semantic and syntactic variability. 

The proposed CNN model achieved its highest performance in the N400-Faces ERP task, with 99% accuracy, precision, and recall, and minimal error rates. It also showed strong and consistent results across the P300 tasks, while performance slightly declined in the N400-Words task and was lowest in the N400-Sentence condition, reflecting challenges in processing complex linguistic structures. A full breakdown of the performance metrics is presented in Table 4.

**Figure 6:**
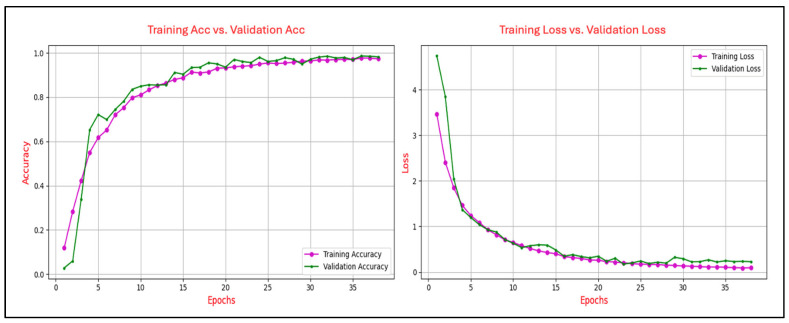
Training and validation accuracy and loss curves for the N400-Faces task.

**Figure 7:**
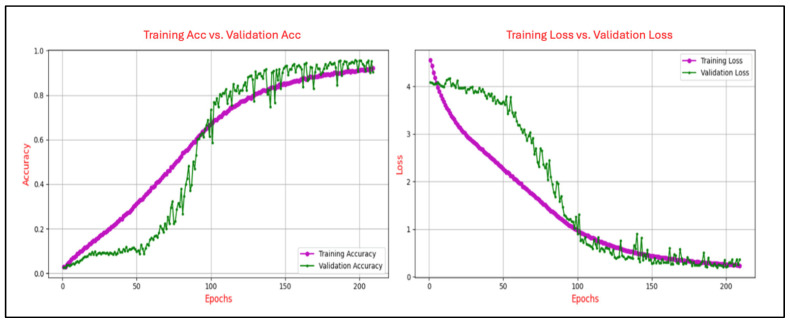
Training and validation accuracy and loss curves for the N400-Words task.

**Figure 8:**
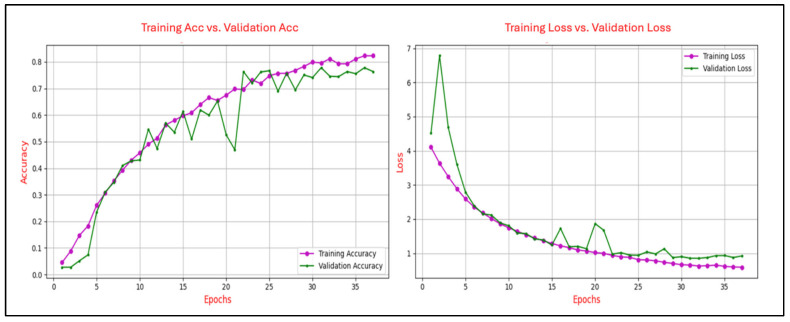
Training and validation accuracy and loss curves for the N400-Sentence task.

**Figure 9:**
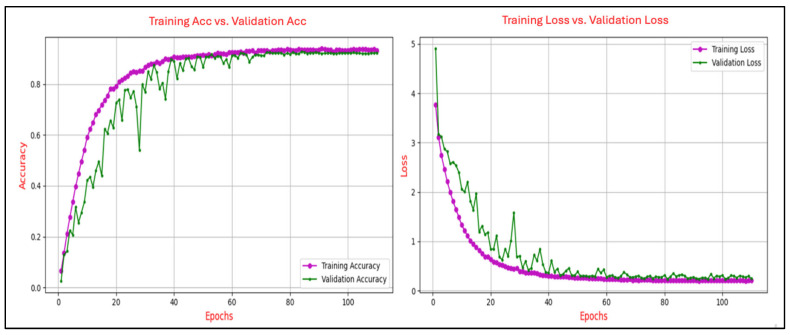
Training and validation accuracy and loss curves for the P300-Selected task.

**Figure 10:**
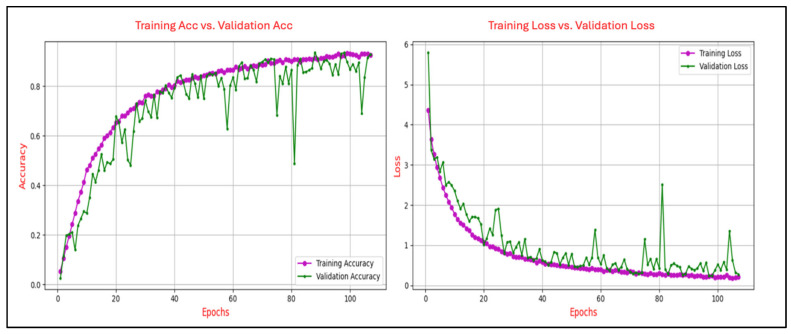
Training and validation accuracy and loss curves for the P300-Assigned ERP task.

Figure 6, Figure 7, Figure 8, Figure 9 and Figure 10 present the evolution of the model’s accuracy and loss across successive epochs for various ERP tasks, evaluated on both the training and validation datasets. These curves reveal an initial upward trend, reflecting the model’s capacity to learn meaningful patterns from the training data.

Figure 6 illustrates the training and validation performance of the CNN model on the N400-Faces task, charting the progression of accuracy (left panel) and loss (right panel) over 38 epochs. The training accuracy exhibits a steady and rapid ascent, nearing 0.97 by the final epoch, while validation accuracy follows a parallel path, ultimately stabilizing at approximately 0.99, indicative of strong generalization and minimal discrepancy between the training and validation phases. Concurrently, training loss declines sharply from an initial value exceeding 3.5 to below 0.2, with validation loss similarly decreasing and converging at around 0.3 after approximately 10 epochs, maintaining stability thereafter. The close correspondence between the training and validation curves in both accuracy and loss substantiates the absence of overfitting. Throughout the training process, no signs of divergence or instability are evident, and the model demonstrates consistent, stable performance across epochs. This pattern reflects the effectiveness of the applied regularization strategies and indicates that the network architecture is well-calibrated to the task.

Figure 7 illustrates the learning dynamics of the CNN model trained on the N400-Words task and depicts the progression of training and validation accuracy and loss over 200 epochs. Training accuracy exhibits a steady upward trend, ultimately reaching approximately 0.68, while validation accuracy follows a similar path, peaking at around 0.73, though moderate fluctuations in later stages suggest sensitivity to variations in validation data. Concurrently, training loss declines sharply from an initial value exceeding 4.0 to below 0.8, with validation loss mirroring this downward trend and stabilizing near 0.6 after approximately 40 epochs.

Figure 8 illustrates the learning dynamics of the CNN model trained on the N400-Sentence task over 38 epochs, showing classification accuracy and loss during both the training and validation phases. Training accuracy demonstrates a steady and continuous improvement, ultimately reaching approximately 0.82, while validation accuracy follows a similar upward path but exhibits noticeable fluctuations, stabilizing around 0.75. These variations suggest sensitivity to the validation data distribution and potential minor inconsistencies in generalization performance. Concurrently, training loss declines consistently from approximately 4.2 to 0.6, reflecting effective model optimization. Validation loss follows a comparable downward trend, albeit with greater variability, converging near 0.8 in the final epochs. Despite these fluctuations, the relatively narrow disparity between training and validation metrics suggests stable learning dynamics with minimal overfitting.

Figure 9 illustrates the progression of training and validation performance metrics for the CNN model on the P300-Selected task, tracing the evolution of accuracy and loss over 110 epochs. Training accuracy exhibits a steady ascent, reaching approximately 0.91 by the final epoch, while validation accuracy follows a closely aligned path, peaking at around 0.90, with both curves demonstrating smooth convergence and minimal divergence. Similarly, training loss declines sharply from an initial value of approximately 3.8 to below 0.2, with validation loss mirroring this trend and stabilizing near 0.2 after 40 epochs.

Figure 10 illustrates the training dynamics of the CNN model on the P300-Assigned task, capturing the evolution of accuracy and loss over 110 epochs. Training accuracy demonstrates a steady upward path, ultimately reaching approximately 0.90, while validation accuracy closely follows this trend, peaking near 0.89 despite intermittent fluctuations, particularly in the mid to late training stages. Concurrently, training loss declines sharply from an initial value exceeding 4.0 to below 0.2, with validation loss following a similar downward trend, stabilizing at around 0.3 despite occasional spikes.

The analysis of the learning curves across different ERP tasks provides valuable insights into the performance and generalization capability of the CNN model. The alignment between training and validation accuracy, coupled with consistent reductions in training and validation loss, demonstrates the model’s effective learning and robust generalization for most tasks. Specifically, for the N400-Faces, N400-Words, P300-Selected, and P300-Assigned tasks, the results indicate good generalization with minimal signs of overfitting, as evidenced by the convergence of training and validation metrics.

In addition to the learning curve analysis, the evaluation of confusion matrices provides a deeper understanding of the CNN model’s classification performance across various ERP tasks. Confusion matrices serve as an essential diagnostic tool, offering a granular breakdown of the model’s predictions and its ability to differentiate between distinct classes. For each ERP task, these matrices detail the distribution of true positives, false positives, true negatives, and false negatives, thereby delivering valuable insights into both overall accuracy and class-specific performance.

**Figure 11:**
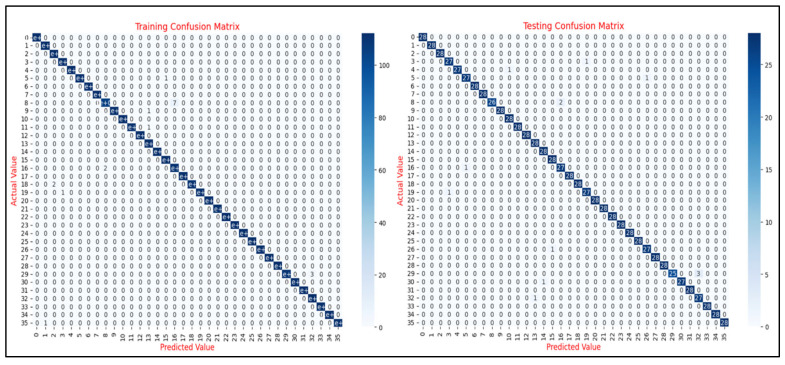
Confusion matrices for the N400-Faces task (training and testing phases).

Figure 11 presents the training and testing confusion matrices for the N400-Faces task, with both matrices exhibiting strong diagonal dominance, indicating high classification accuracy. The training matrix shows near-perfect predictions with minimal misclassifications, while the testing matrix similarly reflects strong generalization with only a few off-diagonal entries. These results confirm the model’s ability to effectively learn and generalize the discriminative patterns of the N400-Faces task, with no substantial signs of overfitting.

The results of the proposed CNN model demonstrate its strong performance across diverse ERP tasks, showcasing its ability to accurately classify EEG data while maintaining robust generalization to unseen samples. The model achieved high accuracy, precision, and recall, with a standout performance on the N400-Faces task (96% accuracy, 97% precision and recall), underscoring its capacity to extract meaningful features from complex neural data. The analysis of the corresponding confusion matrices revealed pronounced diagonal dominance, further attesting to the model’s effective learning and minimal error rates.

### 5.2. Comparison with Classical Classifiers

**Figure 12:**
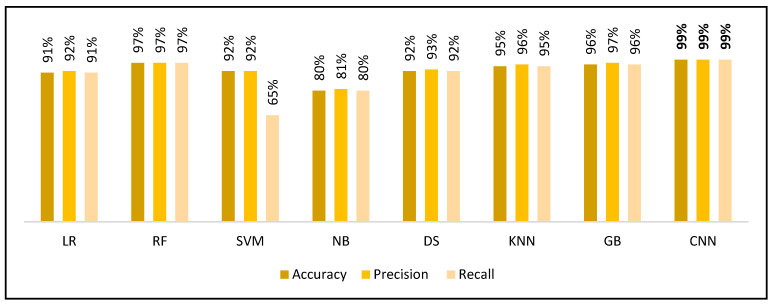
Performance measurements of different classifiers on the N400-Faces task.

To rigorously assess the efficacy of the proposed CNN model across diverse ERP tasks, a comprehensive comparative analysis was conducted utilizing several conventional ML classifiers, including LR, RF, SVM, GB, DT, NB, and DT. The extracted features served as input representations for these classifiers. The evaluation metrics were accuracy, precision, recall FAR, FRR, and EER. Figure 12 illustrates performance measurements of different classifiers on the N400-Faces task.

The CNN model exhibited superior results across all evaluated metrics. Specifically, the CNN achieved 99% accuracy, precision, and recall, outperforming all traditional classifiers. While RF and GB also demonstrate strong performance (both with 97% across metrics), they fell marginally short of CNN’s consistency and peak values. In contrast, models such as SVM and NB showed considerably lower scores, particularly in precision and recall, underscoring their limitations in capturing the nuanced, high-dimensional representations inherent in EEG data.

As shown in Table 5, the CNN model achieved the highest performance across all evaluation metrics on the N400-Faces task, with accuracy, precision, and recall of 99%, and a relatively low error rate (EER = 2%). RF and GB also demonstrated strong performance, with RF achieving an EER of 1.1% and GB closely following at 1.7%. In contrast, NB exhibited the weakest performance across most metrics, with an accuracy of only 80%. These results underscore the superior ability of deep learning approaches, such as CNNs, to capture the complex spatiotemporal dynamics of EEG data, particularly when compared to traditional machine learning classifiers.

Table 6 presents the classification performance across models for the remaining tasks; the N400-Words task, N400-Sentence task, P300-Assigned task, and P300-selected task highlight the comparative effectiveness of different approaches.

For the N400-Words task, the CNN model achieved 90% accuracy, 91% precision, and 90% recall, reflecting strong generalization capabilities in a task characterized by semantic complexity. RF marginally outperformed CNN, reaching 93–94% across all metrics, while GB, KNN, and DS also showed competitive results. In contrast, traditional models, such as LR, SVM, and NB, performed poorly, with accuracies ranging from 19% to 29%, underscoring their limited capacity to model the intricate patterns underlying ERP signals.

For the N400-Sentence task, a paradigm known for its semantic and contextual complexity, the CNN model demonstrated moderate effectiveness, achieving 76% accuracy, 81% precision, and 76% recall, indicating a reasonable capacity to capture sentence-level ERP features despite the task’s inherent difficulty. In comparison, RF achieved the highest performance across all metrics (96%), followed closely by GB and KNN at 93%, showcasing the advantage of ensemble and nonparametric methods. Conversely, traditional models, such as SVM and LR, underperformed significantly, with accuracies as low as 18% and 56%, respectively, highlighting their limited suitability.

For the P300-Assigned task, the CNN model demonstrated strong and consistent results, achieving 92% accuracy, 93% precision, and 92% recall, indicating effective learning and reliable generalization. These results are on par with top-performing models, such as RF and GB, both of which also achieved high performance across all metrics. In contrast, traditional classifiers, such as SVM and LR, performed poorly, with accuracies of 15% and 33%, respectively, underscoring their limited suitability for the P300-assigned task.

For the P300-Selected task, the CNN model demonstrated strong and balanced results, achieving 93% accuracy, 94% precision, and 93% recall. While RF slightly outperformed other models with 95% across all metrics, and GB closely followed at 92%, the CNN model remained highly competitive. Its consistent precision and recall reflect robust generalization and reliable classification across ERP data. In contrast, traditional models, such as LR and SVM, showed limited performance, with accuracies of only 30% and 11%, respectively, highlighting their inadequacy in handling the temporal and cognitive complexity of P300 signals.

Overall, the proposed CNN model exhibited strong and consistent performance across a wide range of ERP tasks, effectively capturing the complex spatiotemporal patterns embedded in EEG signals. Its high levels of accuracy, precision, and recall—most notably in cognitively demanding components, such as N400-Faces—demonstrate the model’s ability to extract and generalize meaningful neural representations. Comparative analyses further underscore CNN’s superiority over conventional machine learning classifiers, which often fall short in handling the nonlinear, high-dimensional nature of EEG data. These results position the CNN framework as a highly effective and scalable approach for EEG-based cognitive and neurophysiological classifications.

### 5.3. Comparison with Previous Studies

The comparative evaluation presented in Table 7 highlights the strengths of the proposed CNN-based model in addressing ERP-based user authentication tasks against previous studies that utilized the same Brainwave Authentication dataset.

The N400-Faces task demonstrated the most significant improvement in classification performance, with the proposed CNN model achieving an accuracy of 99%, surpassing prior approaches. For example, Arias-Carbacos et al. [8] achieved 94% accuracy using AR features and a GNB classifier, while the proposed model’s use of ERP and PSD features with a CNN classifier provided a notable improvement.

For the N400-Words task, the proposed CNN achieved an accuracy of 90%, reflecting a strong performance relative to previous studies. While Arias-Carbacos et al. [28] achieved 88% accuracy using AR features with an SVM classifier, the proposed model’s balanced precision and recall underscore its capacity to generalize effectively despite its inherent complexity. Moreover, Arias-Carbacos et al. [28] reported 88% classification accuracy with SVM, in contrast to the 19% achieved by our SVM model. This pronounced discrepancy can be primarily ascribed to fundamental methodological divergences, most notably in feature extraction strategies. Where our framework employed a hybrid approach combining ERP-derived temporal features and PSD-based spectral components, their methodology relied exclusively on AR coefficients, which are particularly adept at characterizing the stationary dynamics of EEG signals within short temporal segments. Given the inherently subtle nature of the N400-Words paradigm, often characterized by low-amplitude and distributed ERP responses, the AR-based representation may have been more effective in capturing the nuanced cognitive variations relevant to this task.

For the N400-Sentence task, the proposed model achieved 76% accuracy, showcasing moderate performance compared to its results on other tasks. This reflects the challenging nature of this task, which involves intricate linguistic and contextual patterns. Despite this, the model’s performance demonstrates its potential for improvement through refined feature extraction and deeper architectural enhancements. The P300-Selected and P300-Assigned tasks presented in the table indicate that the proposed model significantly outperforms prior CNN-based efforts by Singh et al. [9], which achieved accuracies of 35% and 38%, respectively. The proposed model’s accuracies of 92% for P300-Selected and 93% for P300-Assigned underscore its ability to effectively generalize across ERP paradigms when equipped with ERP and PSD features.

In comparison, the review emphasizes the transformative impact of ERP tasks and PSD features with a CNN classifier in enhancing classification accuracy. The proposed model consistently outperforms traditional methods and prior CNN-based approaches, particularly in the N400-Faces task. These results validate the importance of advanced feature representation and robust model architectures in achieving state-of-the-art performance in EEG-based authentication systems while also highlighting areas for potential improvement in tasks involving greater complexity, such as N400-Sentence.

Moreover, we expanded comparison of previous methodologies that employed ERP for feature extraction using different datasets (Table 8). ERP components have been widely utilized in the literature due to their temporal specificity and reliability in response to task-relevant stimuli. Andronache et al. [29] proposed a similar approach utilizing neural responses to personally meaningful visual stimuli. They extracted spatiotemporal features using a CNN, achieving an identification accuracy of 96.28%.

Wu et al. [7] introduced a biometric authentication technique utilizing rapid visual presentation of self and non-self faces. Their method incorporated hierarchical discriminant component analysis (HDCA) for classification, resulting in a notable accuracy rate of 91.31%. Rathi et al. [18] proposed an EEG-based user authentication method that leverages P300 responses triggered by customized visual stimuli. Features were extracted using ERP analysis, and classification was performed using quadratic discriminant analysis (QDA), achieving an accuracy of 97%.

Collectively, the reviewed studies underscore the efficacy of ERP-based EEG authentication systems, with the majority reporting classification accuracies above 95% and several exceeding 97%. Classifiers such as CNNs and LRs consistently exhibit superior performance, underscoring the discriminative power of temporally aligned ERP features when integrated with advanced learning architectures.

In comparative analysis, TajDini et al. [30] achieved an accuracy of 96.97% using an SVM classifier applied to a general-purpose EEG dataset. In contrast, our implementation of SVM and LR on the N400-Faces task yielded accuracies of 92% and 97%. While the SVM result is comparatively lower, both remain competitive when contextualized against fundamental dataset differences. The datasets used in [30] were not tailored for authentication applications, whereas the present work utilizes a purpose-built dataset specifically designed to capture EEG dynamics in biometric authentication scenarios. This authentication-specific structure, while introducing greater inter-subject variability and cognitive task complexity, contributes to the ecological validity and generalizability of the reported performance.

Some studies did not utilize ERP-aligned data and thus they were not directly comparable to our ERP-centered authentication framework. For example, TajDini et al. [30] reported high accuracy with SVM at 99.06% using a private dataset. The lower performance of the SVM in our pipeline suggests that such conventional classifiers may be will-suited for capturing the complex, non-stationary patterns embedded in ERP responses, particularly when using richer but more variable EEG representations. This further justifies our reliance on CNNs, which consistently outperformed classical methods across all tasks.

Our CNN-based model achieved 99% accuracy, outperforming other CNN implementations, as previously reported in the literature, with accuracies of 97.6% and 96.28% achieved in studies [17,29], thereby affirming the robustness of the proposed architecture in processing ERP–N400 and PSD features for person identification.

### 5.4. Limitations

Several limitations of the proposed framework must be acknowledged. First, the system assumes a stable cognitive and emotional state during both training and authentication. However, EEG signals are inherently sensitive to affective fluctuations such as fatigue, stress, or heightened arousal, which may introduce intra-subject variability and affect model performance. Prior work has shown that emotional states can modulate EEG patterns and alter classification outcomes [31]. As such, the current evaluation does not fully reflect real-world conditions, and future work should explore authentication robustness under varying psychophysiological states.

Additionally, future work may explore embedding EEG-based authentication within cognitively adaptive architectures. Gao et al. [32] demonstrated that multi-domain constraint learning systems based on adaptive cognitive graphs effectively model spatiotemporal dependencies in emotion recognition. Similar mechanisms could enhance EEG-based authentication in diverse applications, including personalized neurosecurity, real-time access control, cognitive state monitoring, adaptive human–computer interaction, and assistive BCI systems.

Second, the Emotiv EPOC+ device, while practical and cost-effective, presents hardware-related limitations. Its relatively low spatial resolution, limited number of electrodes, and susceptibility to noise and motion artifacts may compromise signal quality and restrict the generalizability of findings, particularly in uncontrolled environments. Nonetheless, its ease of use and portability make it a feasible option for biometric applications outside clinical settings.

## 6. Conclusions

This study has undertaken a comprehensive investigation into the design, implementation, and evaluation of an EEG-based authentication system, advancing the field of biometric security by leveraging the unique spatiotemporal and spectral properties of EEG signals. Through the integration of ERP and PSD features within a CNN-based framework, the proposed system achieved robust and superior performance across multiple authentication tasks, surpassing classical machine learning classifiers in terms of accuracy, precision, and recall.

The principal contributions of this study are summarized as follows: (i) The design and implementation of an innovative EEG-based authentication framework, integrating both ERP and PSD features to encapsulate the temporal and spectral dynamics of neural activity, thereby achieving enhanced classification performance relative to conventional methodologies. (ii) The development of an authentication system to investigate the viability of EEG-based authentication paradigms, wherein empirical findings revealed superior accuracy, precision, and recall compared to traditional classifiers across multiple ERP conditions. (iii) A comprehensive comparative evaluation of diverse ERP types within the authentication context, offering a detailed analysis of performance differentials across paradigms—including N400-Faces, N400-Words, P300-Selected, and P300-Assigned—and providing critical insights into their relative efficacy for user authentication applications.

A promising future research direction involves investigating the integration of EEG-based authentication with additional biometric modalities—such as facial recognition, keystroke dynamics, or voice patterns—within a multimodal biometric system. Empirical evidence suggests that combining heterogeneous biometric signals enhances authentication accuracy and resilience against spoofing attacks. Nevertheless, current efforts in this area remain limited in scope. Future work should adopt a more holistic approach, exploring synergistic combinations of facial expressions, EEG signals, keystroke behaviors, and other biometric indicators to construct robust, user-centric, and context-aware authentication systems.

## Figures and Tables

**Table 1:** Common ERP components and their relevance to authentication applications.

ERP	Relevance to Authentication	Cognitive Process	Peak Polarity	Latency (ms)
N100	Reflects initial detection of stimuli, useful in assessing rapid neural responses.	Early sensory processing	Negative	80–120
P200	Indicates early attentional engagement, relevant for evaluating user alertness.	Early attention processes	Positive	150–250
N200	Associated with detection of incongruence or novelty, aiding in distinguishing user responses.	Conflict detection and cognitive control	Negative	180–350
P300	Reflects user attention and cognitive assessment of familiar vs. unfamiliar stimuli, central to user verification.	Attention and stimulus evaluation	Positive	250–500
N400	Indicates recognition of meaningful or contextually relevant information, aiding in validating user-specific responses.	Semantic processing	Negative	300–500
P600	Highlights cognitive processing of complex stimuli, revealing individualized neural response patterns.	Syntactic processing	Positive	500–800

**Table 2:** Dataset description.

Participants	Thirty-Six Participants
Device	14-channel Emotiv EEG headset.
Acquisition protocols	Stimulus-related tasks: Participants were exposed to visual stimuli designed to elicit specific time-locked ERPs, namely the P300 and N400 components.
Stimuli type	Image flashes, including animal, food, and grayscale images, video segments, and individual words.
Duration	Recording lasted for one hour per session.
Paradigm	Oddball paradigm for P300. Semantic processing paradigm for N400.

**Table 3:** Description of the tasks used in the acquisition protocols.

Task	Cognitive Process	Description
P300-Selected	Focus and attentional responses	User self-selected a target stimulus (e.g., a preferred animal image). Target stimuli appeared with low probability among frequent nontargets. Users silently counted target occurrences to maintain an attention state.
P300-Assigned		Identical to P300-Selected but with targets assigned by the experimenter.
N400-Words	Semantic mismatch detection	Semantic Word-Pair Task: the user silently read word pairs with either expected or unexpected relationships.
N400-Sentences		Sentence Context Task: The user is to detect incongruent endings of a sentence. The user read sentences in which the final word was either semantically appropriate or semantically anomalous.
N400-Faces		Picture–Word Matching Task: The user is to detect unfamiliar faces within a sequence of familiar faces. The user viewed an image followed by a word that either matched or mismatched the image.

**Table 4:** CNN model performance across different ERP tasks.

ERP Task	Accuracy	Precision	Recall	FAR	FRR	ERR
P300-Selected	92%	93%	92%	6.6%	7.1%	7.2%
P300-Assigned	93%	94%	93%	5.6%	7.2%	6.4%
N400-Faces	99%	99%	99%	2%	2.1%	2%
N400-Words	90%	91%	92%	8.4%	9.6%	9%
N400-Sentence	76%	81%	76%	19.1%	23.6%	21.4%

**Table 5:** Performance metrics of different classifiers on the N400-Faces task.

Classifier	Accuracy	Precision	Recall	FAR	FRR	ERR
CNN	99%	99%	99%	2%	2.1%	2%
GB	96%	97%	96%	0.1%	3.3%	1.7%
KNN	95%	96%	95%	0.1%	3.9%	2%
DS	92%	93%	92%	2%	7.7%	3.9%
NB	80%	81%	80%	0.5%	0.5%	0.5%
SVM	92%	92%	92%	0.9%	3.2%	3.1%
RF	97%	97%	97%	0.6%	2.1%	1.1%
LR	91%	92%	91%	2%	2.9%	3%

**Table 6:** Performance metrics of different classifiers with different tasks (Acc: Accuracy, Pre: precision, Rec: recall).

Model	P300-Selected Task			P300 Assigned Task			N400-Sentence Task			N400-Words Task		
	Acc	Pre	Rec	Acc	Pre	Rec	Acc	Pre	Rec	Acc	Pre	Rec
LR	30%	30%	30%	33%	32%	33%	56%	60%	56%	29%	32%	29%
RF	95%	95%	95%	92%	92%	92%	96%	96%	96%	93%	94%	93%
SVM	11%	10%	10%	15%	22%	15%	18%	22%	18%	19%	18%	19%
NB	23%	29%	22%	22%	29%	22%	53%	57%	53%	25%	22%	24%
DS	83%	83%	82%	81%	81%	81%	82%	84%	82%	83%	83%	82%
KNN	90%	91%	90%	90%	90%	90%	93%	93%	93%	90%	91%	90%
GB	92%	93%	92%	91%	91%	91%	93%	93%	93%	92%	92%	91%
CNN	93%	94%	93%	92%	93%	92%	76%	81%	76%	90%	91%	90%

**Table 7:** Comparison of previous methodologies used in the Brainwaves Authentication dataset.

Ref.	ERP Task	Extracted Features	Classifier	Accuracy
[28]	N400-Words	AR	SVM	88%
[8]	N400-Faces	AR	GNB	94%
[9]	P300-Selected and P300-Assigned	Not mentioned	CNN	35%
	N400words, P300-Selected, and P300-Assigned	Not mentioned	CNN	38%
**Present work**	**N400-Faces**	**ERP and PSD**	**CNN**	**99%**
	N400-Words			90%
	N400-Sentence			76%
	P300-Selected			92%
	P300-Assigned			93%

**Table 8:** Comparison of ERP-based EEG authentication methodologies in different datasets.

Ref.	Extracted Features	Classifier	Accuracy
[29]	ERP–P300	CNN	96.28%
[7]		HDCA	91.31%
[18]		QDA	97%
**Present work**	**ERP–N400** **and PSD**	**CNN**	**99%**

## Data Availability

The Brainwave Authentication dataset used in this study is available at: https://github.com/kit-ps/bainwave-authentication (accessed on 19 December 2024).
